# CircME1 promotes aerobic glycolysis and sunitinib resistance of clear cell renal cell carcinoma through cis-regulation of ME1

**DOI:** 10.1038/s41388-022-02386-8

**Published:** 2022-07-07

**Authors:** Ming-xiao Zhang, Jia-li Wang, Cheng-qiang Mo, Xiao-peng Mao, Zi-hao Feng, Jia-ying Li, Hai-shan Lin, Hong-de Song, Quan-hui Xu, Ying-han Wang, Jun Lu, Jin-huan Wei, Hui Han, Wei Chen, Hai-ping Mao, Jun-hang Luo, Zhen-hua Chen

**Affiliations:** 1 Department of Urology, The First Affiliated Hospital of Sun Yat-sen University, No. 58, Zhongshan 2nd Road, Guangzhou, 510080 People’s Republic of China; 2 Department of Nephrology, The First Affiliated Hospital of Sun Yat-sen University, No. 58, Zhongshan 2nd Road, Guangzhou, 510080 People’s Republic of China; 3 Department of Urology, Sun Yat-sen University Cancer Center, No. 651, Dongfeng Road East, Guangzhou, 510060 People’s Republic of China; 4 Institute of Precision Medicine, The First Affiliated Hospital of Sun Yat-sen University, No. 58, Zhongshan 2nd Road, Guangzhou, 510080 People’s Republic of China

**Keywords:** Renal cell carcinoma, Oncogenes

## Abstract

Circular RNAs (circRNAs) play critical roles in clear cell renal cell carcinoma (ccRCC). However, their involvement in sunitinib resistance remains largely unknown. Herein, we identified a novel circRNA, named circME1, which contributes to sunitinib resistance development in ccRCC. CircME1 also promoted proliferation, migration, and invasion of ccRCC cells. Further mechanism analysis showed that circME1 interacted with U1 snRNP at the promoter of its parental gene ME1, thereby upregulating the expression of ME1, enhancing aerobic glycolysis of ccRCC, and promoting its malignant phenotype. Furthermore, ME1 specific inhibitor could effectively repress the oncogenic functions of circME1. Taken together, our study demonstrates that the circME1/ME1 pathway is involved in ccRCC progression and sunitinib resistance development, which may be exploited for anticancer therapy.

## Introduction

Renal cell carcinoma (RCC) is a common urinary system malignancy, with a worldwide incidence rate growing 2% per year [1]. Clear cell RCC (ccRCC), the most frequent type of RCC, accounts for 75–80% of total RCC patients. For early stage ccRCC, early diagnosis and surgery can effectively enhance outcomes, however, a 20–40% recurrence rate remains following nephrectomy [2]. For advanced stage ccRCC (20–30%), traditional treatment methods exhibit poor prognosis, and systemic therapy is often the most effective treatment [3, 4]. Sunitinib, a tyrosine kinase inhibitor targeting platelet-derived growth factor, vascular endothelial growth factor, among others, is a widely used first-line treatment medicine for RCC [5]. However, the therapeutic efficiency of sunitinib is significantly limited due to inherent or acquired resistance [6]. Although studies have been performed to investigate underlying mechanisms of sunitinib resistance [7–9], the molecular mechanisms remain unclear and therefore require further study.

Circular RNAs (circRNAs), a class of small noncoding RNAs with a configuration of covalent single-stranded loop, are derived from exon skipping or back-splicing of precursor mRNA [10]. With the development and wide application of high throughput sequencing in recent years, more and more circRNAs have been identified to function in both pathological and physiological processes, including cancer progression and metastasis [11]. CircRNAs play critical roles in multiple processes. They can regulate the activities of miRNAs [12, 13], regulate gene expression at both splicing and transcription levels [14, 15], function as encoded proteins after being translated [16], or interact with RNA-binding proteins [17]. These findings suggest that circRNAs play crucial roles in a series of fundamental processes and can thereby serve as potential clinical markers for treatment of diseases, such as cancer.

CircRNAs have been implicated in a series of biological processes of cancers, including tumor growth, metastasis, metabolic reprogramming and development of therapeutic resistance [18–21]. However, whether and how circRNAs contribute to the development of sunitinib resistance in RCC treatment remains largely unknown. Herein, we identified a novel circRNA, named circME1, which is correlated with development of sunitinib resistance and poor prognosis of RCC patients. Meanwhile, we demonstrated that circME1 promotes ccRCC aerobic glycolysis and malignancy. Further investigation showed that circME1 interacts with U1 snRNP and promotes transcription of its parental gene ME1 in cis, thereby promoting tumor development and sunitinib resistance. Our study suggests that circME1 may serve as a promising biomarker to predict sunitinib resistance and therapeutic target of ccRCC.

## Results

### CircME1 is associated with sunitinib resistance and poor survival of ccRCC

**Fig. 1:**
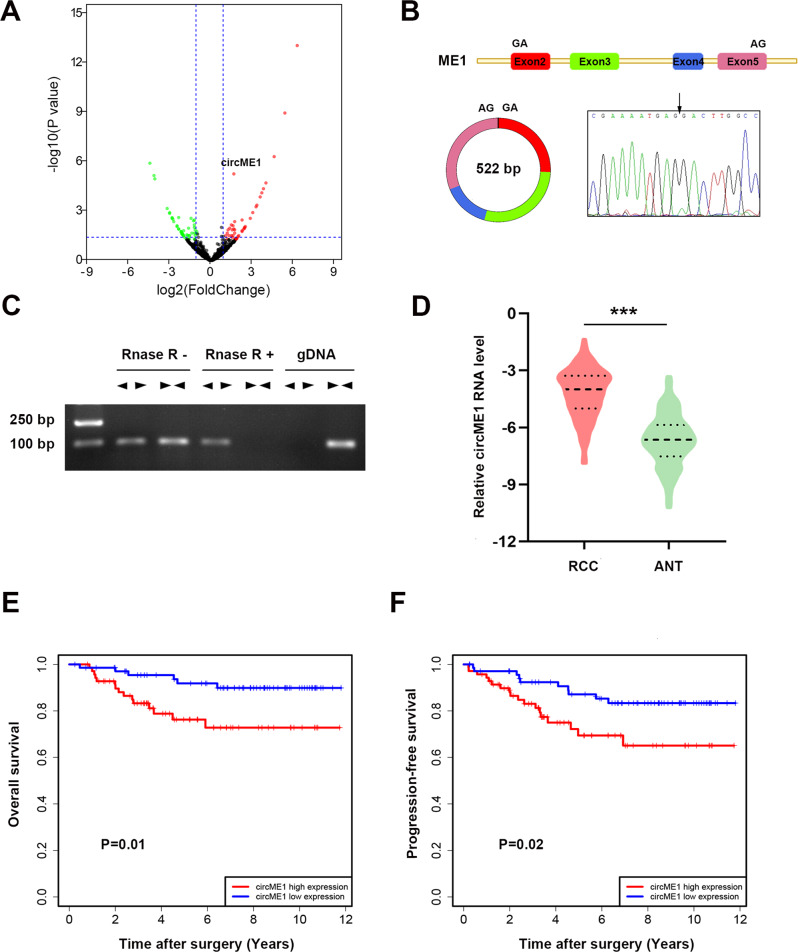
Circular RNA identification using RNA-seq and characteristics of circME1 in ccRCC. **A** Volcano plot of up-regulation and down-regulation of circRNAs in sunitinib-resistant cells. **B** The circME1 expression was determined by Sanger sequencing. The back-splicing site of circME1 is marked by black arrowhead. **C** The circME1 was identified by qPCR and electrophoresis. Divergent and convergent primers were used to amplify the back-splicing site and linear ME1 mRNA, respectively. CircME1 can only be amplified in cDNA and is resistant to RNase R. **D** The relative circME1 level in 140 pairs of ccRCC and matched normal tissues was measured using qPCR. **E**, **F** Survival analysis showing that high-circME1 expression was associated with poor OS and PFS in our ccRCC cohort. ***p < 0.001.

We sought to screen potential sunitinib resistance-related circRNAs using RNA-seq, and we found that circME1 has the highest expression level and is one of the most significantly up-regulated circRNAs in sunitinib-resistant cells (Fig. 1A). CircME1 is derived from the ME1 gene and generated by its exon2 to exon5 via back-splicing. Sanger sequencing indicated that the exon2 and exon5 of ME1 gene are back-spliced to form the closed loop structure (Fig. 1B). To verify the expression of circME1 in ccRCC cells, divergent primers and convergent primers were designed for the back-spliced form of circME1 and linear transcript respectively. The cDNA and genomic DNA were amplified and analyzed by nucleic acid electrophoresis (Fig. 1C). Furthermore, qPCR results of our ccRCC cohort showed that the expression of circME1 dramatically increased in ccRCC tissues (Fig. 1D). Then, the ccRCC patients were classified into high and low-circME1 expression groups with median level of circME1 as cut-off value. The high-circME1 expression group exhibited enhanced tumor size, lymph node metastasis, distant metastasis and Fuhrman nuclear grade compared to low-circME1 expression group (Supplementary Table 1). Moreover, the Kaplan–Meier survival analysis demonstrated that in our ccRCC cohort, high-circME1 expression was related to poor overall survival and progression-free survival (Fig. 1E, F). Taken together, these results suggest that circME1 might play critical roles in tumorigenesis and progression of ccRCC, therefore is a potential prognostic marker in ccRCC.

### Silencing circME1 suppresses cell proliferation and tumor growth of sunitinib-resistant ccRCC cells in vitro and in vivo

**Fig. 2:**
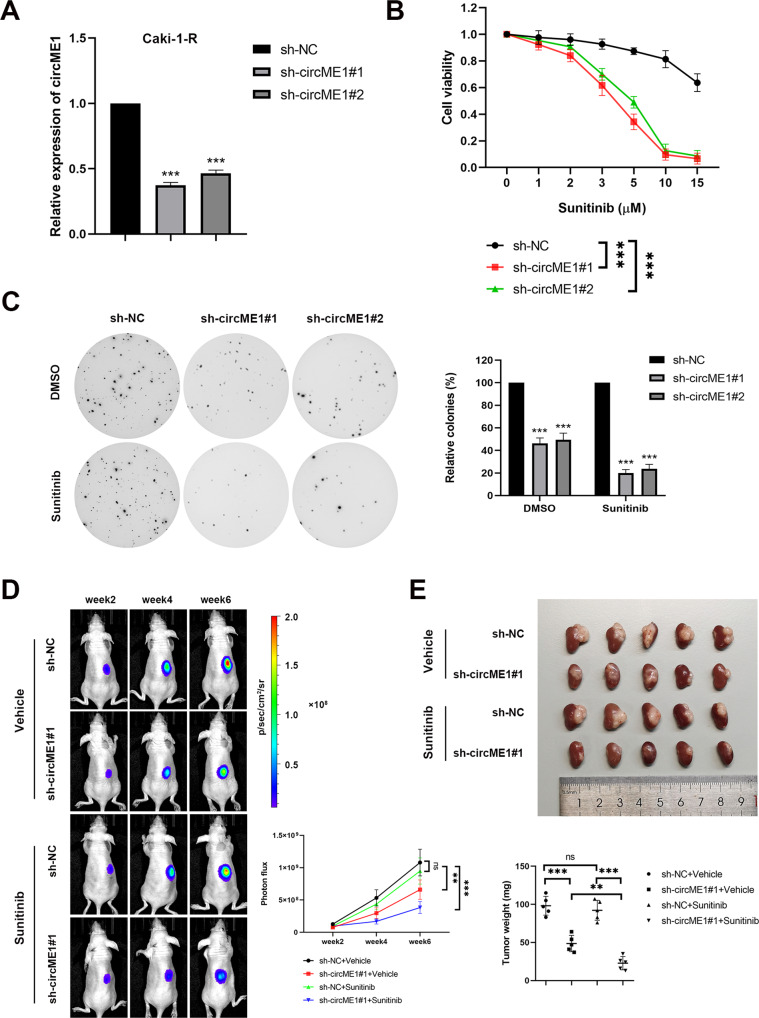
CircME1 promotes sunitinib resistance of ccRCC cells in vitro and in vivo. **A** CircME1 was silenced in Caki-1-R cells by two different shRNAs, and the silencing efficiency was determined by qPCR. **B** CCK8 assay showing that the growth of Caki-1-R cells was suppressed by circME1 knockdown upon sunitinib treatment. **C** Soft agar colony formation assay showing that circME1 knockdown not only inhibited sunitinib resistance with sunitinib treatment (2 µM), but also repressed proliferation and colony formation without sunitinib treatment. **D**, **E** Knockdown of circME1 significantly suppressed the growth and sunitinib resistance of xenograft in vivo. Nude mice with orthotopic tumor were treated with sunitinib (40 mg/kg) or vehicle. The representative bioluminescence images of orthotopic tumors (left panel) and the corresponding statistical analyses (right panel) are shown in **D**. Other images are shown in Supplementary Fig. S1. The images of gross tumors (upper panel) and the final tumor weights (lower panel) are shown in **E**. ns not significant, **p < 0.01, ***p < 0.001.

To further investigate the functions of circME1 in sunitinib-resistant ccRCC, Caki-1-R cells were transfected with short hairpin RNAs (shRNAs) against circME1. As showed in Fig. 2A, circME1 expression in Caki-1-R cells was significantly decreased by circME1 shRNAs. In addition, silencing circME1 in Caki-1-R cells remarkably suppressed cell proliferation upon sunitinib treatment (Fig. 2B). Moreover, knockdown of circME1 also markedly repressed anchorage-independent growth of Caki-1-R cells treated with either sunitinib or DMSO (Fig. 2C), suggesting circME1 could also act as an oncogene in ccRCC cells. To further explore the functions of circME1 in vivo, circME1-silencing Caki-1-R cells or counterpart control Caki-1-R cells were inoculated into BALB/c nude mice, followed by treatment with sunitinib or vehicle. As shown in Fig. 2D, E, knockdown of circME1 significantly suppressed tumor growth upon treatment of either sunitinib or vehicle, and circME1-silencing group treated with sunitinib displayed the lowest tumor growth rate among all groups. These results indicate that circME1 may promote sunitinib resistance of ccRCC in vitro and in vivo.

### CircME1 enhances proliferation of ccRCC cells in vitro

**Fig. 3:**
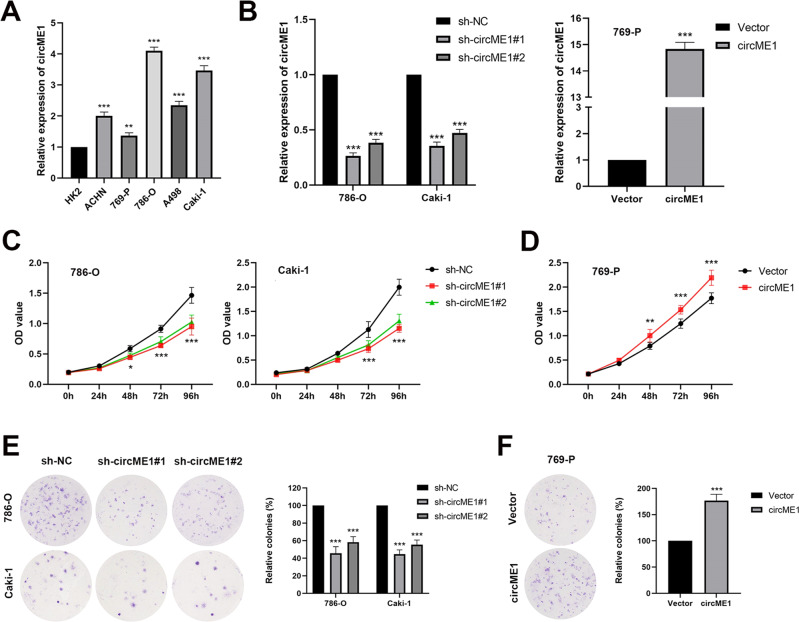
CircME1 promotes ccRCC proliferation in vitro. **A** CircME1 expression levels in different ccRCC cell lines and immortalized proximal tubule epithelial cells (HK2). **B** CircME1 was silenced in 786-O and Caki-1 cells using two different shRNAs (left panel) and overexpressed in 769-P cells (right panel). CCK8 and colony formation assays showing that the proliferation and colony formation of ccRCC cells were suppressed by circME1 knockdown (**C**, **E**), but promoted by circME1 overexpression (**D**, **F**). *p < 0.05, **p < 0.01, and ***p < 0.001.

As circME1 may act as an oncogene in ccRCC cells, we next explore the roles of circME1 in progression of ccRCC. We first examined circME1 expression in a series of ccRCC cell lines (ACHN, 769-P, 786-O, A498 and Caki-1), and also immortalized proximal tubule epithelial cells (HK2). Consistent with clinical results, compared with HK2 cells, circME1 was evidently up-regulated in ccRCC cell lines (Fig. 3A). We next further verify the oncogenic functions of circME1 using silencing/overexpression experiments. Results showed that silencing circME1 in 786-O and Caki-1 cells dramatically suppressed while overexpressing circME1 in 769-P cells significantly promoted cell proliferation and colony formation (Fig. 3B–F). These results suggest that circME1 may function to enhance ccRCC proliferation in vitro.

### CircME1 promotes migration and invasion of ccRCC cells in vitro and in vivo

**Fig. 4:**
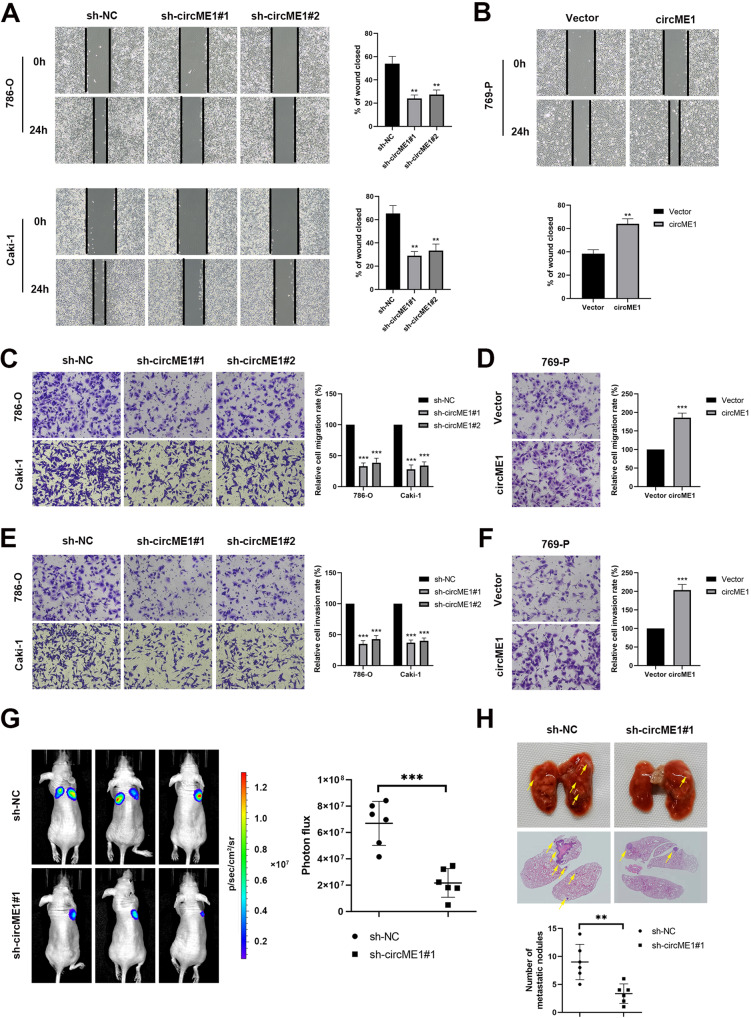
CircME1 enhances ccRCC cell migration and invasion in vitro and metastasis in vivo. **A**, **B** Wound-healing assay showing that circME1 knockdown significantly suppressed while circME1 overexpression promoted mobility of ccRCC cells. The representative images and quantitative analysis results are shown. The migration and invasion of ccRCC cells were inhibited by knockdown of circME1 (**C**, **E**) and promoted by overexpression of circME1 (**D**, **F**). The migrated or invaded cells were counted in five random fields and the migration or invasion rate was normalized to the control groups. **G** The ccRCC lung metastasis was significantly inhibited by circME1 depletion as evaluated in a lung metastasis mouse model. The representative bioluminescence images of lung metastases (left panel) and statistical analysis results (right panel) are shown. Other images are shown in Supplementary Fig. S2A. **H** The nude mice injected with circME1-silencing 786-O cells exhibited fewer and smaller lung metastases. The representative images of gross and HE stained lungs are shown (upper panel). The pulmonary metastatic nodules were counted under a microscope and analyzed (lower panel). The pulmonary metastatic nodules are marked with arrowheads. Other images are shown in Supplementary Fig. S2B. **p < 0.01, ***p < 0.001.

We next explored the effect of circME1 on cell motility. We found that knockdown of circME1 significantly attenuated migration of 786-O and Caki-1 cells in wound-healing assay, while circME1 overexpression promoted 769-P cell migration (Fig. 4A, B). Further, in transwell migration assay, compared with 769-P cells with ectopic expression of circME1, knockdown of circME1 remarkably suppressed migration of 786-O and Caki-1 cells (Fig. 4C, D). Similarly, in matrigel invasion assay, knockdown of circME1 significantly inhibited invasion of 786-O and Caki-1 cells compared with 769-P cells (Fig. 4E, F). Furthermore, an in vivo tumor metastasis mouse model was used to test the effect of circME1 on ccRCC metastasis in vivo. Results showed that compared with control group, circME1 depletion significantly attenuated the lung metastases of 786-O cells (Fig. 4G, H). These results demonstrated that circME1 may be able to enhance migration and invasion of ccRCC cells, thereby promoting ccRCC progression and metastases.

### CircME1 promotes its parental gene ME1 expression

**Fig. 5:**
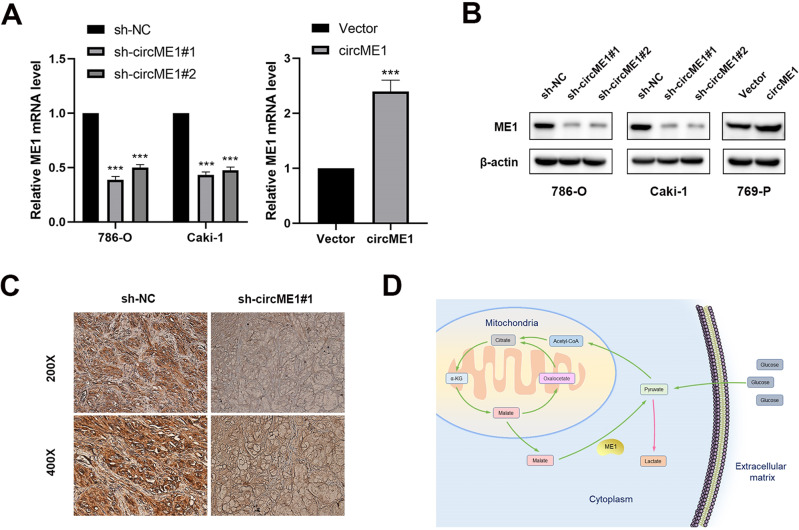
CircME1 promotes expression of its parental gene ME1. **A** qPCR results showed that the mRNA level of ME1 was decreased after knockdown of circME1 and increased after overexpression of circME1. **B** Western blot analysis showed that knockdown and overexpression of circME1 changed the protein level of ME1. **C** IHC analysis showed that the ME1 protein level was remarkably reduced in xenografts generated from circME1-silencing cells. **D** The schematic diagram of ME1 in glucose metabolism. ***p < 0.001.

To analyze the specificity of shRNAs of circME1, we also simultaneously tested the expression of its parental gene, ME1. The qPCR results showed that the expression of ME1 was also decreased in circME1-silencing 786-O and Caki-1 cells (Fig. 5A, left panel). Interestingly, the expression of ME1 was also increased in circME1-overexpressing 769-P cells (Fig. 5A, right panel). To further explore the effect of circME1 on ME1, we examined ME1 protein expression in ccRCC cells with silenced circME1 (786-O and Caki-1 cells) or overexpressed circME1 (769-P cells). Results showed that knockdown of circME1 markedly down-regulated ME1 protein in both 786-O and Caki-1 cells. In contrast, overexpression of circME1 remarkably up-regulated ME1 protein in 769-P cells (Fig. 5B). Moreover, compared with control, IHC staining of tumors derived from circME1-deficient Caki-1-R cells also exhibited lower level of ME1 protein (Fig. 5C). ME1 is a multifunctional oncogenic gene involved in aerobic glycolysis [22, 23], NADPH production [24] and lipid metabolism [25]. A schematic diagram of ME1 functions in glucose metabolism is shown in Fig. 5D. Collectively, these data demonstrated that there is a cis-acting regulation between circME1 and its parental gene ME1. Then we tested whether circME1 could regulate the glucose metabolism in ccRCC via ME1.

### CircME1 promotes ccRCC aerobic glycolysis in vitro

**Fig. 6:**
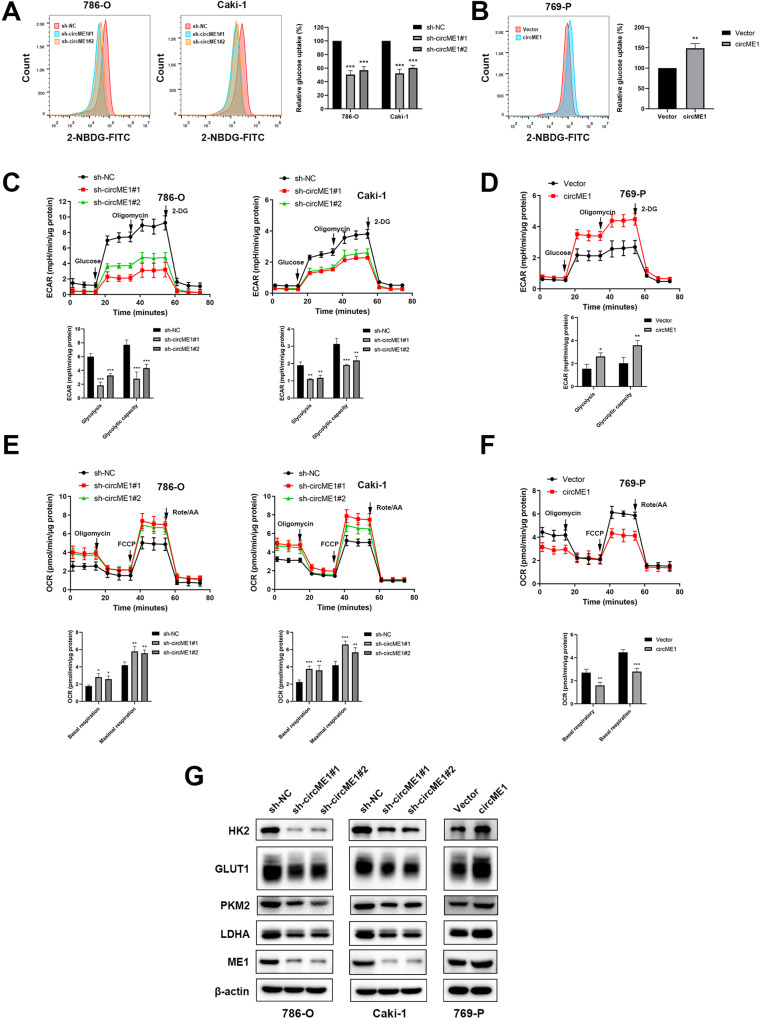
CircME1 promotes ccRCC glycolysis in vitro. The relative glucose uptake of ccRCC cells was inhibited by knockdown of circME1 (**A**) and promoted by overexpression of circME1 (**B**). **C**, **D** Knockdown of circME1 markedly impaired glycolysis and glycolytic capacity of ccRCC cells, while overexpression of circME1 enhanced them. ECAR was measured using the Seahorse analyzer. Glucose, oligomycin and 2-DG were sequentially injected at the indicated time points. **E**, **F** Knockdown of circME1 increased OCR, while overexpression of circME1 decreased it. **G** Knockdown of circME1 remarkably decreased the key glycolytic enzymes while overexpression of circME1 significantly increased the expression of these glycolytic enzymes in ccRCC cells. *p < 0.05, **p < 0.01, ***p < 0.001.

Since ME1 plays critical roles in aerobic glycolysis and the expression level of ME1 is correlated with circME1, we hypothesized that circME1 may affect the glycolysis process in ccRCC cells. To verify this hypothesis, we analyzed glucose uptake, glycolytic flux and oxidative phosphorylation in ccRCC cells with down-regulated or up-regulated circME1. Results showed that knockdown of circME1 decreased glucose uptake in 786-O and Caki-1 cells (Fig. 6A), while overexpression of circME1 increased glucose uptake in 769-P cells (Fig. 6B). We next evaluated glycolytic flux by testing extracellular acidification rate (ECAR). Results showed that shRNAs induced knockdown of circME1 decreased glycolytic flux in 786-O and Caki-1 cells. Glycolysis and glycolytic capacity were both decreased by circME1 knockdown (Fig. 6C). In contrast, overexpression of circME1 enhanced glycolysis in 769-P cells (Fig. 6D). Moreover, we measured oxygen consumption rate (OCR) to explore the effect of circME1 on oxidative phosphorylation. Results showed that oxygen consumption was enhanced by circME1 knockdown while suppressed by its overexpression (Fig. 6E, F). We also tested the protein levels of a series of key glycolytic enzymes including HK2, GLUT1, PKM2 and LDHA. Results showed that knockdown of circME1 significantly reduced while overexpression of circME1 dramatically increased the expression of these glycolytic enzymes in ccRCC cells (Fig. 6G). In summary, the data suggest that circME1 can promote ccRCC aerobic glycolysis in vitro.

### CircME1 promotes the expression of its parental gene ME1 via interacting with U1 snRNP

**Fig. 7:**
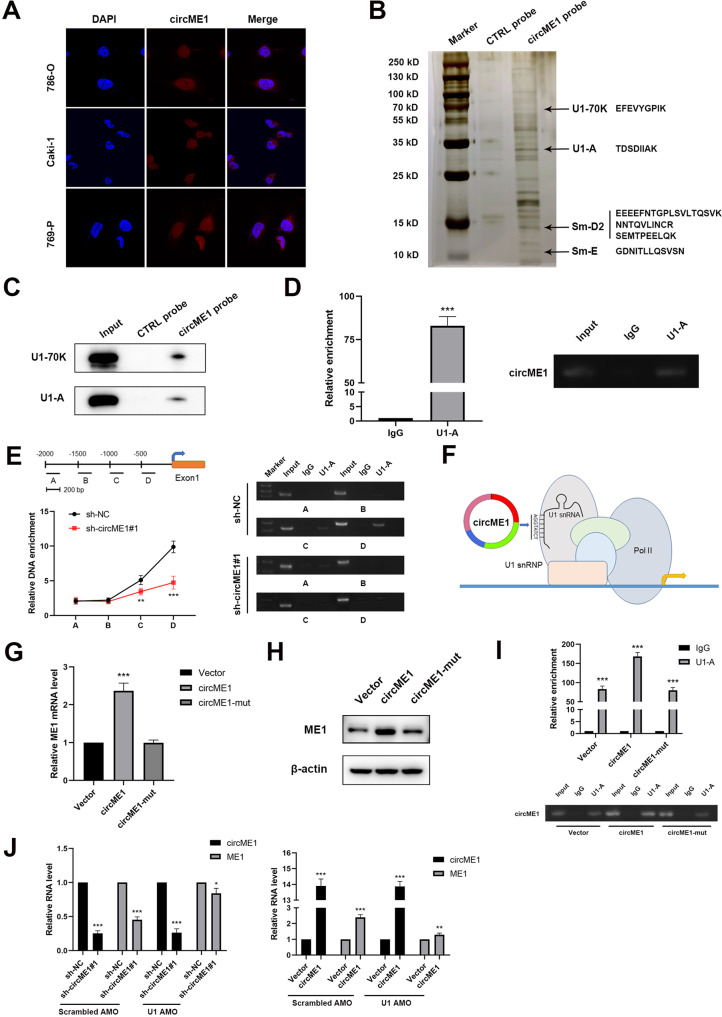
CircME1 exerts cis-regulatory effect via interacting with U1 snRNP. **A** CircME1 was abundantly distributed both in nucleus and cytoplasm as demonstrated by RNA-FISH with CY3-labeled circME1 probe and DAPI labeled nuclei. **B**, **C** U1-70K, U1-A, Sm-D2 and Sm-E (protein components of U1 snRNP) were identified as circME1-interacting proteins by RNA pull-down assay. The proteins pulled down by circME1 or CTRL probes were subject to SDS-PAGE and silver staining (**B**). U1-70K and U1-A were pulled down by circME1 as demonstrated by Western blot analysis (**C**). **D** CircME1 was markedly enriched in U1-A-immunoprecipitated RNA as demonstrated by RIP assay with IgG as negative control (left panel). Electrophoresis analysis result of qPCR products of immunoprecipitated RNA is shown (right panel). **E** U1-A bound to ME1 promoter (around −500 bp–−300 bp) in 786-O cells as demonstrated by ChIP assay. Knockdown of circME1 decreased the enrichment level of this DNA sequence (left panel). Electrophoresis analysis result of the ChIP products is shown (right panel). **F** The schematic diagram of cis-regulatory effect of circME1 on its parental gene. **G**, **H** qPCR and Western blot analyses showing that overexpression of circME1 containing mutant binding site of U1 snRNP failed to increase the expression level of ME1. **I** CircME1 containing mutant binding site of U1 snRNP failed to be enriched in U1-A-immunoprecipitated RNA as demonstrated by RIP assay (upper panel). Electrophoresis analysis result of the qPCR products of immunoprecipitated RNA is shown (lower panel). **J** Blockage of U1 snRNA with U1 AMO abrogated the cis-regulatory effect of circME1 at mRNA level of ME1 as demonstrated by qPCR assay.

To further elucidate the underlying mechanism of circME1 in cis-regulating its parental gene ME1, RNA-FISH assay was conducted and results showed that circME1 was located both in nucleus and cytoplasm (Fig. 7A). Next, we used RNA pull-down assay coupled with mass spectrometry to identify circME1-associated proteins (Supplementary Tables 2 and 3). Among the 151 proteins which were specifically pulled down by circME1 probe, U1-70K, U1-A, Sm-D2 and Sm-E were particular interesting candidates (Fig. 7B and Supplementary Table 4). These proteins are important components of U1 snRNP which has been reported to interact with some circRNAs through U1 snRNA at the promoters of parental genes to enhance gene expression [15, 26]. Considering that U1-A and U1-70K are particle-specific proteins for U1 snRNP, we further validated the results using independent RNA pull-down assay following by western blot. Results showed that U1-70K and U1-A were pulled down by circME1 probe (Fig. 7C). In addition, RIP assay was conducted and results showed that circME1 was dramatically enriched in U1-A-immunoprecipitated RNAs (Fig. 7D). Therefore, the findings above indicate that circME1 exerts its cis-regulatory effect by binding to U1 snRNP in ccRCC cells. ChIP assay was then used to explore whether U1 snRNP could bind to the promoter of ME1 and identify the possible binding site(s). As shown in Fig. 7E, U1-A tended to bind to the ME1 promoter at around −500 to −300 bp, while silencing circME1 significantly reduced the enrichment level of U1-A within this DNA sequence.

To further dissect the cis-regulatory effect of circME1 on its parental gene, we next identify possible binding site(s) of U1 snRNA on circME1 RNA, and we found that there was only one putative binding site, which was similar to the binding site of U1 snRNA with only 2 bases difference. Thus, circME1 may interact with U1 snRNA through this putative site (AGGTATCT) located in the junction of exon 2 and 3, thereby enhancing the transcription of ME1 (Fig. 7F). Next, we constructed a circME1 mutant plasmid in which this putative binding site was changed from AGGTATCT to GTTGCCAA. qPCR and Western blot results showed that only overexpression of wild type circME1 increased the expression level of ME1 (Fig. 7G, H). The results of RIP assay using 786-O cells transfected with plasmids encoding circME1 and circME1 mutant (circME1-mut) revealed that circME1 containing mutant binding site for U1 snRNA could not be enriched in U1-A-immunoprecipitated RNAs (Fig. 7I). U1 antisense morpholino (AMO) was then used to block the binding site on U1 snRNA, and the qPCR results showed that blocking the binding site could abolished the cis-regulatory effect of circME1 on its parental gene at mRNA level (Fig. 7J).

### CircME1 enhances tumor growth and metastasis by promoting ME1 expression

To elucidate whether circME1 exerts its oncogenic function through ME1, we overexpressed ME1 in circME1-silencing 786-O cells and also treated circME1-overexpressing 769-P cells with ME1 inhibitor. As shown in Supplementary Fig. S3A, ectopic expression of ME1 significantly abolished inhibition of 786-O proliferation induced by circME1 silencing. Moreover, in vitro cell motility assays all showed that overexpression of ME1 impaired the inhibitory effect of circME1 knockdown on migration and invasion of ccRCC cells (Supplementary Fig. S3B, C). Similarly, inhibition of ME1 attenuated the promotive effect of circME1 overexpression on proliferation and motility of ccRCC cells (Supplementary Fig. S3D–F). Taken together, these results indicate that circME1 enhances ccRCC tumor growth and metastasis mainly through ME1.

### CircME1 promotes sunitinib resistance and aerobic glycolysis of ccRCC cells via enhancing ME1 expression

To further dissect the effects of circME1/ME1 pathway, we overexpressed ME1 in circME1-silencing Caki-1-R cells. Results showed that the inhibitory effect of circME1 knockdown on proliferation and anchorage-independent growth of Caki-1-R cells were significantly attenuated by ME1 overexpression (Supplementary Fig. S4A, B). 2-NBDG Uptake Assay was then performed and results showed that ectopic expression of ME1 or treatment with ME1 inhibitor remarkably abrogated circME1 silencing- or overexpression-induced the inhibitory or promotive effects on glucose uptake of ccRCC cells (Supplementary Fig. S4C, D). Furthermore, ME1 overexpression or treatment with ME1 inhibitor abrogated circME1 silencing or overexpression-induced inhibition or promotion of glycolysis and glycolytic capacity of ccRCC cells (Supplementary Fig. S4E, F). Meanwhile, circME1 silencing or overexpression-induced promotive or inhibitive effects on oxidative phosphorylation level of ccRCC cells were markedly attenuated by overexpression of ME1 or treatment with ME1 inhibitor (Supplementary Fig. S4G, H). Taken together, these findings suggest that circME1-ME1 pathway plays critical roles in sunitinib resistance development and aerobic glycolysis of ccRCC cells.

## Discussion

Anti-angiogenic drugs, such as sunitinib, have been developed for RCC therapy, based on the frequent inactivation of VHL in RCC [27]. Despite the effective anti-angiogenic and anti‐tumor activities of sunitinib, treatment of RCC with sunitinib usually failed after 6–15 months due to the development of drug resistance [6]. Therefore, it is urgently required to elucidate the underlying mechanism behind sunitinib resistance and identify novel targets or strategies to prevent or delay sunitinib resistance development. Herein, we identified a novel circRNA, named circME1, which exhibited a high expression in sunitinib-resistant ccRCC cells, and is functionally required for sunitinib-resistant phenotype. Meanwhile, circME1 was found to be significantly up-regulated in ccRCC tissues and correlated with a shorter survival time. Silencing and overexpression experiments showed that circME1 promoted proliferation, migration and invasion of ccRCC cells. Our results also showed that circME1 exerts these oncogenic effects via cis-regulation of its parental gene ME1, thereby regulating glucose metabolism of ccRCC cells. Taken together, our results showed that circME1 plays a critical role in ccRCC tumor progression and development of sunitinib resistance.

Recently, increasing studies have focused on function and mechanism studies of circRNAs in many cancers, such as renal cell cancer [28, 29], prostate cancer [30, 31], breast cancer [32, 33], lung cancer [34], colorectal cancer [35] and hepatocellular carcinoma [36]. However, the biological functions of most circRNAs remain largely unexplored. Understanding the mechanisms behind sunitinib resistance development can help explore novel therapy strategies to overcome or attenuate sunitinib resistance. Herein, we found that circME1 could physically interact with U1 snRNP. Several circRNAs have been reported to hold U1 snRNP through interaction with U1 snRNA to form circRNA-U1 snRNP complexes, which could further enhance gene expression via binding to Pol II transcription complex at the promoters of parental genes [15]. In this study, we demonstrated that circME1 could enhance the expression of its parental gene ME1 in cis through interaction with U1 snRNP at the promoter of ME1. Furthermore, both mutation of the putative U1 snRNP binding site on circME1 and blocking U1 snRNA binding with AMO could abolish this cis-regulation.

Malic Enzyme 1 (ME1) is a multifunctional enzyme, which catalyzes malate conversion to pyruvate and mediates NADPH generation from NADP. ME1 has been demonstrated to function in lipogenesis, glycolysis, and NADPH homeostasis [22–25], however, its functions in ccRCC remain unknown. Herein, we identified ME1 as a downstream target of circME1. Meanwhile, we found that circME1 significantly promoted aerobic glycolysis of ccRCC cells via ME1. Aerobic glycolysis, also known as Warburg effect, is involved in tumor progression and sunitinib resistance development [37–39]. We also found that circME1 exerted diverse oncogenic functions in ccRCC tumor progression and sunitinib resistance development, which could be dramatically repressed by ME1 inhibitor. Taken together, these results suggest that circME1/ME1 pathway is closely correlated with progression, sunitinib therapy response and prognosis of ccRCC.

In summary, our study demonstrated for the first time that circME1 could act as a valuable biomarker for ccRCC prognosis and sunitinib response prediction. Our findings reveal a potential mechanism underlying the functions of circME1 for promoting sunitinib resistance in ccRCC via cis-regulation of its parental gene ME1, which highlights a promising strategy to enhance clinical therapeutic efficacy of sunitinib with ME1 specific inhibitors in high-circME1 expression ccRCC patients.

## Materials and methods

### RNA sequencing

Caki-1 and Caki-1-R cells were lysed with Trizol reagent (Invitrogen, CA, USA) and RNA was isolated according to the standard method. RNA sequencing was performed by BGI Genomics Co., Ltd (Shenzhen, China). In total, 3 μg of total RNA was treated with DNase I to degrade DNA presenting in RNA samples. Then, ribosomal RNA was removed using the Ribo-off rRNA Depletion Kit and linear RNA was removed using RNase R. Purification was performed using Agencourt RNAClean XP magnetic beads. All other steps were performed according to the manufacturer’s protocols. The library was quality and quantitated in two methods: check the distribution of the fragments size using the Agilent 2100 bioanalyzer, and quantify the library using BMG (OMEGA). Finally, the Qualified libraries were sequenced pair end on the BGISEQ-500 or MGISEQ-2000 (BGI-Shenzhen, China). The RNA sequencing results are shown in Supplementary Table 5.

### Cell culture and clinical samples

ACHN, A498, Caki-1, 786-O, 769-P and HK2 cell lines were purchased from the Chinese Academy of Science. All cell lines were authenticated by short tandem repeat profiling and tested negative for mycoplasma contamination. Caki-1 cells were cultured in McCoy’s 5A medium. 786-O and 769-P cells were cultured in RPMI-1640 medium. A498, ACHN, and HK2 were cultured in Dulbecco’s Modified Eagle medium. All the media were supplemented with 10% fetal bovine serum, and all the cells were cultured in a 37 °C humidified 5% CO_2_ incubator. The ccRCC and matched adjacent normal tissue samples were collected from 140 patients from December 2007 to December 2018 at Sun Yat-sen University Cancer Center (Guangzhou, China), and the clinicopathological information of these patients is shown in Supplementary Table 1. The informed consent was obtained from each patient. This study was approved by Ethical Committee of Sun Yat-sen University Cancer Center (Guangzhou, China).

### Plasmid construct and shRNA transfection

Plasmids encoding circME1 and circME1 mutant (circME1-mut) were purchased from Geneseed (Guangzhou, China). For construction of plasmids encoding circME1, the full length of human circME1 cDNA was amplified by PCR using the following primers: circME1-forward: CGGAATTCTAATACTTTCAGGACTTGGCCTTTACCCTGGAA and circME1-reverse: CGGGATCCAGTTGTTCTTACCTCATTTTCGGTTCCCACATC. Then, the circME1 sequence incorporated with EcoR I and BamH I sites was ligated into pLC5-ciR vector after restriction digests. For construction of plasmids encoding circME1-mut, the circME1 cDNA was amplified by PCR using the following two primer pairs respectively: circME1-forward: CGGAATTCTAATACTTTCAGGACTTGGCCTTTACCCTGGAA and circME1-inR1: TTGGAGATCCATTAAGAGATTGGCAACGTCAAAGTCAGAGTTCAGATGCTCGAAATTT (product 1: 159 bp); circME1-inF1: GCATCTGAACTCTGACTTTGACGTTGCCAATCTCTTAATGGATCTCCAAGATAGAA and circME1-reverse: CGGGATCCAGTTGTTCTTACCTCATTTTCGGTTCCCACATC (product 2: 412 bp). These two kinds of products were purified and amplified by PCR using circME1-forward and circME1-reverse primers to get the circME1-mut amplicons. Then, the circME1-mut sequence incorporated with EcoR I and BamH I sites was ligated into pLC5-ciR vector after restriction digests. Plasmids encoding ME1 was purchased from Vigenebio (Jinan, China). shRNAs against circME1 and corresponding control were obtained from the GeneChem Company (Shanghai, China). 293T cells were transfected with circME1 overexpression plasmids and sh-circME1 plasmids, and the supernatants were harvested and concentrated to prepare lentivirus. 786-O, Caki-1 and 769-P cells were then transfected with lentivirus, and 2 μg/ml puromycin was used for selection for 2 weeks to obtain stable transfected cell lines. The shRNA sequences are shown in Supplementary Table 6.

### 2-NBDG uptake assay

Glucose uptake was measured using 2-NBDG. Cultured ccRCC cells were washed with PBS and then incubated in glucose-free RPMI-1640 medium containing 2-NBDG (20 μM) for 2 h, followed by flow cytometry analysis.

### Measurement of cellular metabolism

The ECAR and OCR of ccRCC cells were examined using a Seahorse XF extracellular flux analyzer (Agilent, CA, USA). ccRCC cells were seeded in Agilent Seahorse XF96 plates (8000 cells/well for 786-O and 769-P and 15,000 cells/well for Caki-1) and cultured overnight, followed by a 1-h equilibration with XF Base media in a 37 °C incubator lacking CO_2_, and Seahorse assay was carried out next. After sequential addition of glucose (10 mM), oligomycin (1 µM), and 2-DG (50 mM), ECAR was measured for assessment of glycolysis stress. For test of MitoStress, compounds were added sequentially as follows: oligomycin (1.5 µM), FCCP (1 µM for 786-O and Caki-1, 0.5 µM for 769-P), and rotenone/antimycin A (0.5 µM). Metabolic data were obtained and normalized to protein concentration determined using BCA protein assay (Thermo, MA, USA).

### In vivo mouse experiments

All animal experiments were in compliance with ethical regulations and approved by the Institutional Animal Care and Use Committee of Sun Yat-sen University.

For orthotopic xenograft tumor model, 20 male BALB/c nude mice (4 weeks old) were randomly distributed into two groups, and injected with circME1-silencing Caki-1-R-luc cells or counterpart control Caki-1-R-luc cells (1 × 10^6^/mouse) orthotopically into right renal subcapsule. After 2 weeks, the nude mice were randomized for oral administration of sunitinib (40 mg/kg/day) or vehicle. After 6 weeks, mice were sacrificed, and kidneys with xenograft tumors were harvested. The weight of orthotopic xenograft was obtained by subtracting the contralateral kidney weight from the total weight of kidney with xenograft tumor.

For lung metastasis assay, 12 male BALB/c nude mice (4 weeks old) were randomly distributed into two groups and intravenously injected with circME1-silencing 786-O-luc cells or counterpart control 786-O-luc cells (1 × 10^6^/mouse). After 6 weeks, mice were sacrificed, lungs were harvested, and the pulmonary metastatic nodules were counted.

For tumor growth and metastasis analysis, nude mice bearing Caki-1-R-luc or 786-O-luc cells were injected with D-Luciferin, and bioluminescence signals were analyzed using IVIS Spectrum.

We did not perform sample size calculations, and determined the sample size according to literature reports as well as our experience. Randomization was conducted to determine the grouping of experimental mice. All experimental mice were numbered by body weight and allocated to different experimental groups according to random number table. No sample was excluded from the analysis, and no blinding was done.

### Fluorescence in situ hybridization (FISH) assay

FISH assay was conducted using a FISH Kit (GenePharma, Shanghai, China) by following the manufacturer’s instruction. CY3-labeled circME1 probe was synthesized by Geneseed (Guangzhou, China). Cell nuclei were stained with DAPI, and analyzed using confocal microscopy.

### RNA pull-down and RNA immunoprecipitation (RIP) assays

A Pierce™ Magnetic RNA-protein pull-down kit (Thermo Fisher Scientific, MA, USA) was used for RNA pull-down. Briefly, biotin-labeled circME1 junction probe and control probe were incubated with streptavidin magnetic beads at RT for 30 min, and then 100 µg 786-O cell protein extract was incubated with RNA-beads mixture at 4 °C overnight. The RNA-binding proteins were separated by SDS-PAGE, visualized by silver staining, and analyzed by mass spectrometry.

RIP assay was conducted using a Magna Nuclear RIP™ (Cross-Linked) Nuclear RNA-Binding Protein Immunoprecipitation Kit (Millipore, MA, USA). Briefly, cultured 786-O cells were incubated with 0.3% formaldehyde at RT for 10 min for crosslinking, followed by quenching with glycine for 5 min. Cells were then lysed and sonicated to harvest sheared cross-linked chromatin. Protein A/G beads were incubated with 5 μg of anti-U1-A (Abcam, catalog number: ab166890) or anti-IgG antibodies at RT for 30 min, and the sheared cross-linked chromatin was added and incubated at 4 °C overnight. The immunoprecipitated RNA was extracted, purified, and analyzed by qPCR.

### Chromatin immunoprecipitation (ChIP) assay

ChIP assay was conducted using a SimpleChIP Plus Enzymatic Chromatin IP Kit (Cell signaling technology, MA, USA) by following the manufacturer’s instructions. Briefly, 10 μg of anti-U1-A or anti-IgG antibodies were incubated with cross-linked and digested chromatin at 4 °C overnight, and protein G magnetic beads (30 µl) were then added and incubated for 2 h. The immunoprecipitated DNA was purified and analyzed by qPCR. The primers used are listed in Supplementary Table 6.

### ME1 inhibitor

ME1 inhibitor (CAS No.: 522649-59-8) was purchased from MedChemExpress (NJ, USA) and suspended in DMSO.

### Transfection of antisense moroholino oligonucleotide (AMO)

Antisense moroholino oligonucleotides (AMOs), including scrambled AMO and U1 AMO, were synthesized by Gene Tools (OR, USA). AMO treatment was conducted using electroporation with a Nucleofector system (Lonza, Basel, Switzerland). In total, 8 h after AMO transfection, cells were collected for downstream experiments. The sequence of the U1 AMO is listed in Supplementary Table 6.

The methods of sunitinib-resistant RCC cell model construction, gDNA and RNA extraction, RNase R treatment, cDNA synthesis, Quantitative real-time PCR, Western blot, Cell counting kit-8 and colony formation assays, Soft agar assays, Wound-healing, transwell migration and matrigel invasion assays, and immunohistochemistry analysis are shown in Supplementary Information. All experiments were replicated three times.

### Statistical analysis

All statistical analyses were conducted using R (version 3.6.3). Two-tailed Student’s *t* test was used for intergroup comparison. The correlation between circME1 expression and clinicopathological parameters was explored using chi-square test. The coefficient of variation was determined and comparisons between groups were conducted. *p* < 0.05 was considered statistically significant.

## Supplementary information

Supplementary information

Supplementary data

Supplementary Table 2

Supplementary Table 3

Supplementary Table 4

Supplementary Table 5
