# Supplementary material for: CircME1 promotes aerobic glycolysis and sunitinib resistance of clear cell renal cell carcinoma through cis-regulation of ME1
Source: Oncogene. 2022 Jul 7;41(33):3979–90. doi: 10.1038/s41388-022-02386-8 (PMC9374592; doi:10.1038/s41388-022-02386-8)
Supplement: Supplementary file 1 — Supplementary information [file 41388_2022_2386_MOESM1_ESM.docx]

**Supplementary Fig. S1: The bioluminescence images of orthotopic tumors.** The bioluminescence images of other nude mice in 4 groups are shown.

**Supplementary Fig. S2: CircME1 promotes ccRCC metastasis in vivo.** A, The bioluminescence images of lung metastases of other nude mice in 2 groups are shown. B, The images of gross and HE stained lungs of other nude mice in 2 groups are shown. The pulmonary metastatic nodules are marked with arrowheads.

**Supplementary Fig. S3: circME1 enhances tumor growth and metastasis via ME1.** A-C, ME1 overexpression abrogated inhibition of ccRCC cell proliferation (A), migration and invasion (B, C) led by circME1 silencing. D-F, Treatment with ME1 inhibitor (50 µM) abolished enhancement of ccRCC cell proliferation (D), migration and invasion (E, F) led by circME1 overexpression. Data are shown as mean ± SD. *P < 0.05, **P < 0.01, and ***P < 0.001.

**Supplementary Fig. S4: circME1 enhances sunitinib resistance and glycolysis of ccRCC cells via ME1.** A, B, ME1 overexpression abolished circME1 silencing-induced suppression of Caki-1-R cell viability treated with sunitinib for 72 h (A), and anchorage-independent growth treated with sunitinib (2 µM) (B). C, D, ME1 overexpression or ME1 inhibitor (50 µM) treatment abolished the effect of circME1 silencing or overexpression on relative glucose uptake of RCC cells. E-H, ME1 overexpression abolished circME1 silencing-induced inhibition of glycolysis and glycolytic capacity (E) and enhancement of oxidative phosphorylation of ccRCC cells (G). Treatment with ME1 inhibitor (50 µM) abolished circME1 overexpression-induced enhancement of glycolysis and glycolytic capacity (F) and suppression of oxidative phosphorylation of ccRCC cells (H). Data are shown as mean ± SD. *P < 0.05, **P < 0.01, and ***P < 0.001.

**Supplementary Table 1:** Association of circME1 expression with clinicopathological information in ccRCC.

**Supplementary Table 2:** Proteins pulled down by CTRL probe.

**Supplementary Table 3:** Proteins pulled down by circME1 probe.

**Supplementary Table 4:** Proteins specifically pulled down by circME1 probe.

**Supplementary Table 5:** circRNA sequencing results.

**Supplementary Table 6:** Primers, shRNA sequences and U1 AMO sequence used in this study

**Supplementary materials and methods**

**Sunitinib-resistant RCC cell model**

A sunitinib-resistant RCC cell xenograft mouse model was developed. Male BALB/c nude mice (4-5 weeks old) were injected with Caki-1 cells (5 × 10^6^ cells/100 µL/mouse), and when the tumor volume reached 200 mm^3^ (the tumor volume was calculated as Length × Width × Width / 2), the first-generation mice were daily administrated with sunitinib (40 mg/kg/day) or vehicle orally for 4 weeks, followed by a 2-week break. The mice were sacrificed after 6 weeks, and tumors were harvested and cut into 1 mm^3^ tumor blocks. The second-generation mice were grafted with these 1 mm^3^ tumor blocks, followed by administration of sunitinib or vehicle. After sacrifice of the third-generation mice, tumors were isolated, washed with cold PBS containing 2% penicillin-streptomycin and levofloxacin (100 µg/mL), and cut into small blocks. The tumor blocks were then digested in the McCoy's 5A medium containing 0.2% Collagenase 4, 0.01% Hyaluronidase and 0.002% DNase I (Stemcell, Canada) at 37°C for 45 min with continuous shaking, followed by centrifugation at 300 g. The cells were then re-suspended and plated in a 6-well plate at a concentration of 7 × 10^5^ cells/well. Fibroblasts were removed through reduplicative adherence assay. Sunitinib-resistant Caki-1 cell was named as Caki-1-R.

**gDNA and RNA extraction**

Genomic DNA (gDNA) was isolated using a gDNA isolation kit (Sangon, Shanghai, China), and total RNA was isolated using Trizol reagent (Invitrogen, CA, USA) by following the manufacturer’s instructions.

**RNase R treatment, cDNA synthesis, and PCR**

Total RNA (2 μg) was incubated with 3 U/μg of RNase R (Epicenter Technologies, WI, USA) at 37°C for 30 min, and purified with a RNeasy MinElute cleaning Kit (Qiagen, Germany). Next, the RNA was first reverse-transcribed to cDNA, and then subject to PCR using GoTaq Green Master Mix (Promega, WI, USA), followed by electrophoresis analysis. The primer sequences are shown in Supplementary Table 6*.*

**Quantitative real-time PCR (qPCR)**

SYBR Green Pro Taq HS premix (Accurate Biology, Changsha, China) was used for qPCR analysis with Beta-actin as internal control. The PCR primers used are shown in Supplementary Table 6.

**Western blot**

ccRCC cells were lysed and equal amounts of cell lysates were subject to SDS-polyacrylamide gel electrophoresis (PAGE) separation, and then transferred onto polyvinylidene fluoride (PVDF) membranes. After a 1-h blockage with 5% fat-free milk at room temperature (RT), the membranes were incubated with the following primary antibodies at 4°C overnight: anti-PKM2 antibody (Catalog number: GB11392, 1:1000) obtained from Servicebio (Wuhan, China), anti-ME1 antibody (Catalog number: 16619-1-AP, 1:1000) obtained from Proteintech (Wuhan, China); anti-Beta-actin (Catalog number: 4970, 1:1000) and anti-HK2 antibodies (Catalog number: 2867, 1:1000) obtained from Cell signaling Technology (MA, USA); anti-LDHA (Catalog number: ab101562, 1:1000) and anti-GLUT1 antibodies (Catalog number: ab115730, 1:5000) obtained from Abcam (Cambridge, UK). The membranes were then incubated with horseradish peroxidase (HRP)-conjugated secondary antibody for 1 h at RT, followed by signal detection using a western blot substrate kit (Tanon, Shanghai, China).

**Cell counting kit-8 (CCK-8) and colony formation assays**

For CCK-8 assay, cells were plated in a 96-well plate (1,500 cells/well) and cultured for 96 h. CCK-8 solution (MedchemExpress, NJ, USA) was added (10 µL/well) and incubated at 37°C for 2 h, followed by absorbance measurement at 450 nm. For colony formation assay, cells were plated in a 6-well plate (1,000 cells/well) and cultured for two weeks, and then the colonies were fixed with 4% paraformaldehyde, stained with 0.1% crystal violet, and counted (> 50 cells).

**Soft agar assays**

1.2% agar was mixed with McCoy's 5A medium and added to 6-well dish. RCC cells were suspended in McCoy's 5A medium containing 20% FBS with Sunitinib (2 µM) or DMSO, mixed with equal volume of 0.7% agar, and seeded in the pre-coated 6-well dish (5×10^3^ cells/well). After 4 weeks culture, colonies were stained with iodonitrotetrazolium chloride (1 μg/mL) and counted.

**Wound-healing, transwell migration and matrigel invasion assays**

For wound-healing assay, wound was generated by scratching cell confluent monolayer using a 200 µL pipette tip. The wound width was measured to determine cell movement. For transwell migration and invasion assays, serum-free medium (100 µL) containing cells was seeded into the upper chamber (50,000 cells/well) of 24-well transwell chamber (Corning, NY, USA) with or without pre-coated matrigel (Corning, NY, USA), and medium containing 10% FBS was added to the lower chamber. The cells were fixed, stained, the non-migrated cells were removed, and the migrated cells were counted under a light microscope.

**Immunohistochemistry (IHC) analysis**

Tumor paraffin sections were baked for 1 h at 65°C, deparaffinized in xylene, and rehydrated with graded ethanol. The sections were then subject to antigen retrieval using EDTA antigen retrieval solution, and blocked with 5% bovine serum albumin solution. The sections were incubated with anti-ME1 antibody (Proteintech, catalog number: 16619-1-AP, 1:100) overnight at 4°C next, and then incubated with HRP-conjugated goat anti-rabbit IgG (ZSGB-BIO, Beijing, China) at RT for 20 min. DAB substrate solution (ZSGB-BIO, Beijing, China) was then added, followed by counterstaining with hematoxylin, dehydrating and mounting.
