# Supplementary material for: CircME1 promotes aerobic glycolysis and sunitinib resistance of clear cell renal cell carcinoma through cis-regulation of ME1
Source: Oncogene. 2022 Jul 7;41(33):3979–90. doi: 10.1038/s41388-022-02386-8 (PMC9374592; doi:10.1038/s41388-022-02386-8)
Supplement: Supplementary file 2 — Supplementary data [file 41388_2022_2386_MOESM2_ESM.pdf]

Supplementary Fig. S1: The bioluminescence images of orthotopic tumors

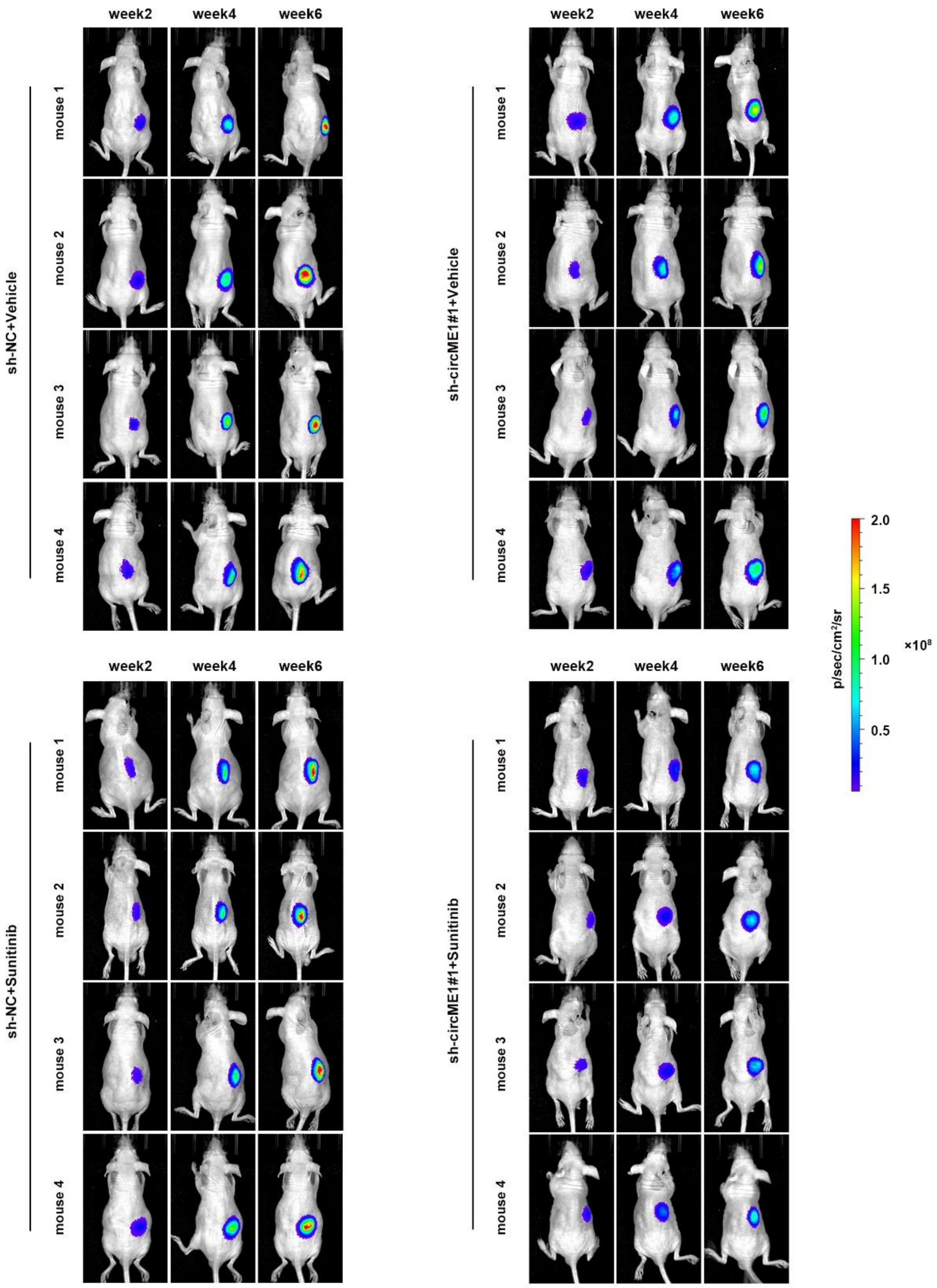

Supplementary Fig. S2: CircME1 promotes ccRCC metastasis in vivo

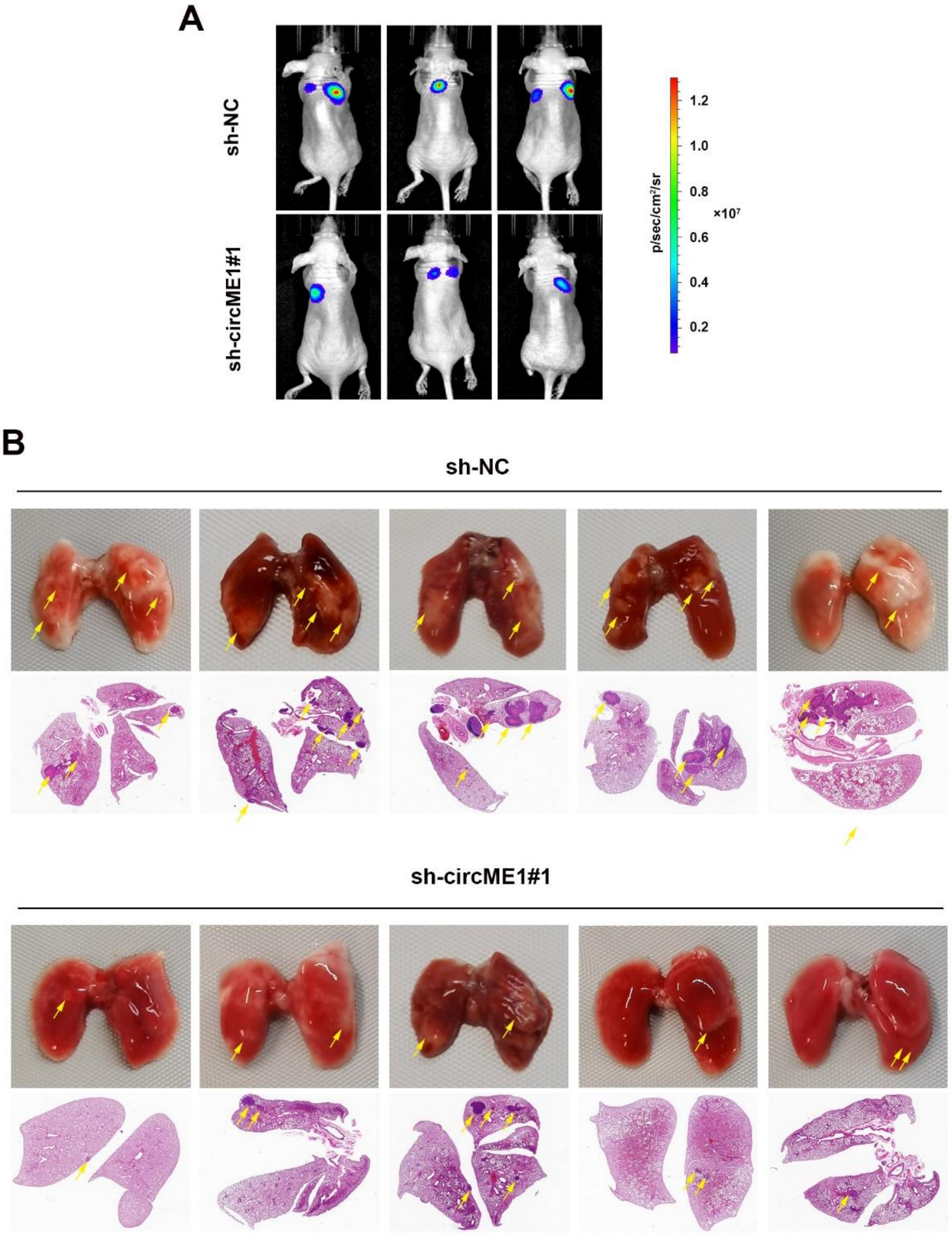

Supplementary Fig. S3: circME1 enhances tumor growth and metastasis via ME1.

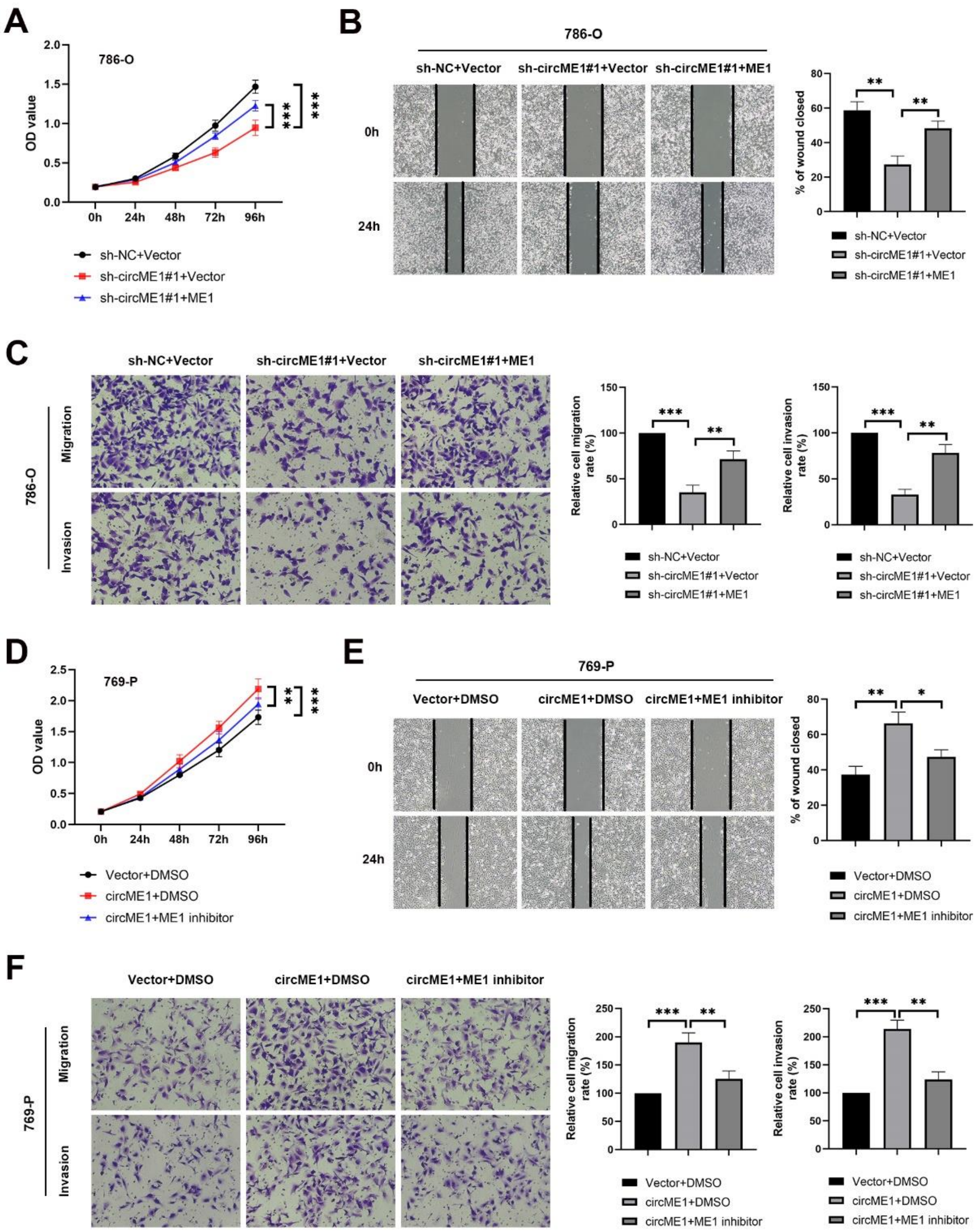

Supplementary Fig. S4: circME1 enhances sunitinib resistance and glycolysis of ccRCC cells via ME1.

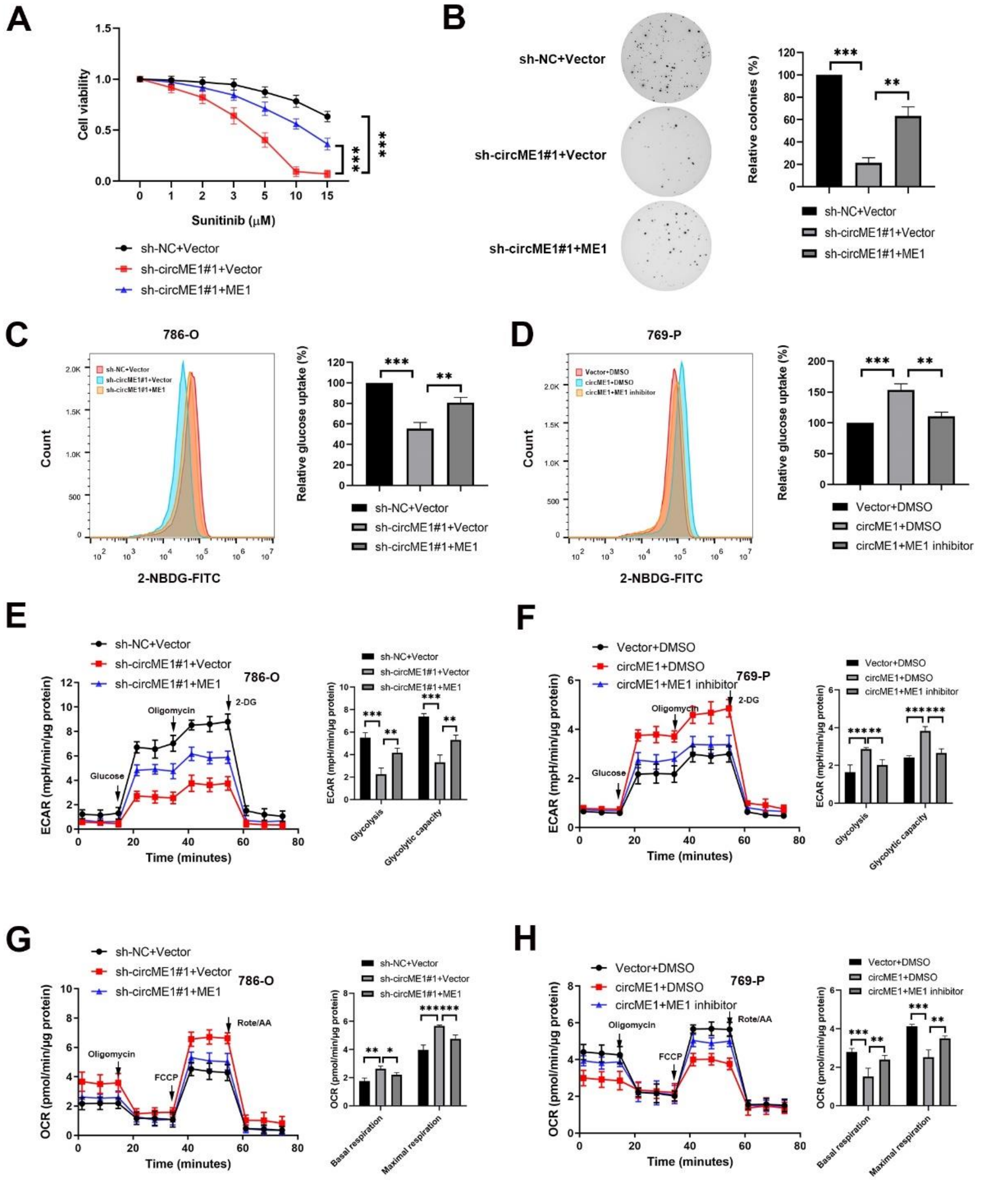

Supplementary Table 1: Association of circME1 expression with clinicopathological information in ccRCC.

**Variable**	Total（%）	circME1 expression		P value
		Low	High	
**Sex**				
Male	96 (68.6%)	47 (33.6%)	49 (35.0%)	0.716
Female	44 (31.4%)	23 (16.4%)	21 (15.0%)	
**Age (Median, range)**	53 (18-80)	50.5 (26-80)	54.5 (18-80)	0.403
**T stage**				
1	82 (58.6%)	47 (33.6%)	35 (25.0%)	0.029
2	25 (17.9%)	12 (8.6%)	13 (9.3%)	1/2 VS 3/4
3	26 (18.5%)	9 (6.4%)	17 (12.1%)	
4	7 (5.0%)	2 (1.4%)	5 (3.6%)	
**N stage**				
0	127 (90.7%)	67 (47.9%)	60 (42.8%)	0.042
1	13 (9.3%)	3 (2.1%)	10 (7.2%)	
**M stage**				
0	129 (92.1%)	68 (48.6%)	61 (43.5%)	0.028
1	11 (7.9%)	2 (1.4%)	9 (6.5%)	
**AJCC stage**				
I	78 (55.7%)	45 (32.1%)	33 (23.6%)	0.006
II	19 (13.6%)	11 (7.9%)	8 (5.7%)	I/II VS III/IV
III	28 (20.0%)	11 (7.9%)	17 (12.1%)	
IV	15 (10.7%)	3 (2.1%)	12 (8.6%)	
**Fuhrman grade**				
1	27 (19.3%)	12 (8.6%)	15 (10.7%)	0.025
2	82 (58.6%)	48 (34.3%)	34 (24.3%)	1/2 VS 3/4
3	25 (17.8%)	8 (5.7%)	17 (12.1%)	
4	6 (4.3%)	2 (1.4%)	4 (2.9%)	

Supplementary Table 6: Primers, shRNA sequences and U1 AMO sequence used in this study

Primers	
circME1-F	GGGAACCGAAAATGAGGACTT
circME1-R	ACCTGGATCTCCTGACTGTT
ME1-F	TGGTGGTGCATTCTCAGAACA
ME1-R	GAACATTCTGCTTTGCTAGTTGGAT
ACTB-F	CCTGGCACCCAGCACAAT
ACTB-R	GGGCCGGACTCGTCATAC
ChIP-A-F	AATGGCTTCTTCAGCTAGGC
ChIP-A-R	TCTCCCTCAAAGGCAACATT
ChIP-B-F	CAATGAAAGGTGTCATTTAACTTCA
ChIP-B-R	TGTTAACTTTGCCTTATCAGAAAGA
ChIP-C-F	GGAAGGCCTGAAAGACTGCT
ChIP-C-R	TCAAGGTGTTAGGGTGCAAA
ChIP-D-F	ATCCCCCTAGGTGTCTGAGC
ChIP-D-R	GGCATGCCTAAACGCAGT
shRNA (target sequence)	
sh-circME1#1	AACCGAAAATGAGGACTTG
sh-circME1#2	ACCGAAAATGAGGACTTGG
U1 AMO	GGTATCTCCCCTGCCAGGTAAGTAT
